# Supplementary material for: Establishment and Characterization of a Primary Fibroblast Cell Culture from the Amazonian Manatee (Trichechus inunguis)
Source: Animals (Basel). 2024 Feb 22;14(5):686. doi: 10.3390/ani14050686 (PMC10931340; doi:10.3390/ani14050686)
Supplement: Supplementary file 1 [file animals-14-00686-s001.zip › animals-2788774-supplementary.pdf]

Establishment of a Primary Continuous Fibroblast Cell Culture from the Amazonian Manatee (*Trichechus inunguis*)

Tavares et al.

Supplementary material S1:

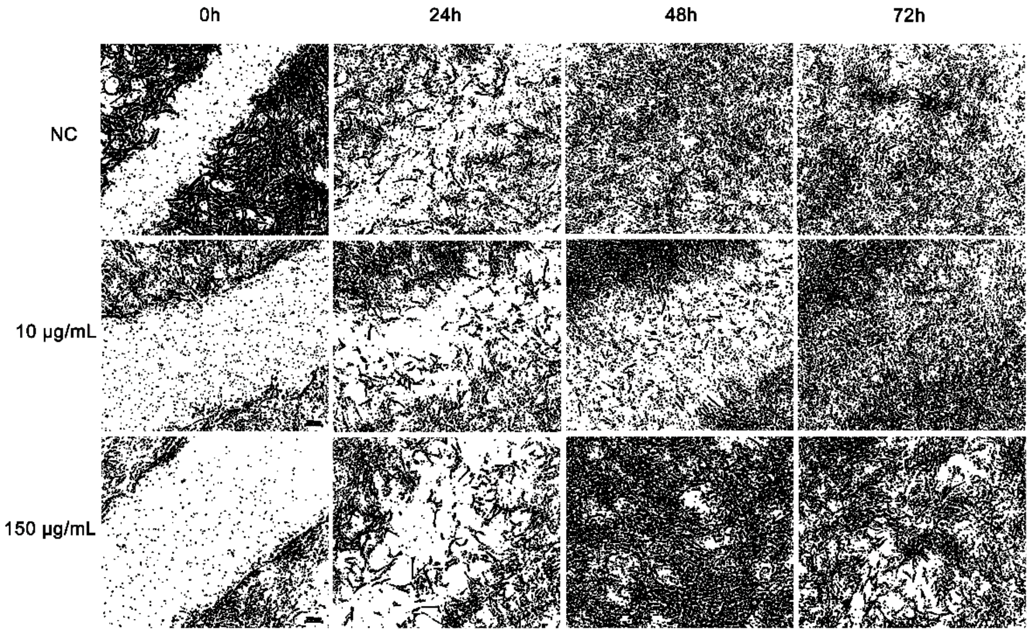

Figure S1: Images from the TINsf cell scratch test were analyzed using Image J 1.54d software to measure the healing area at different concentrations of andiroba seed oil (10 µg/mL and 150 µg/mL) at 24-hour intervals up to 72 hours; scale bar 100 µm.

Supplementary material S2:

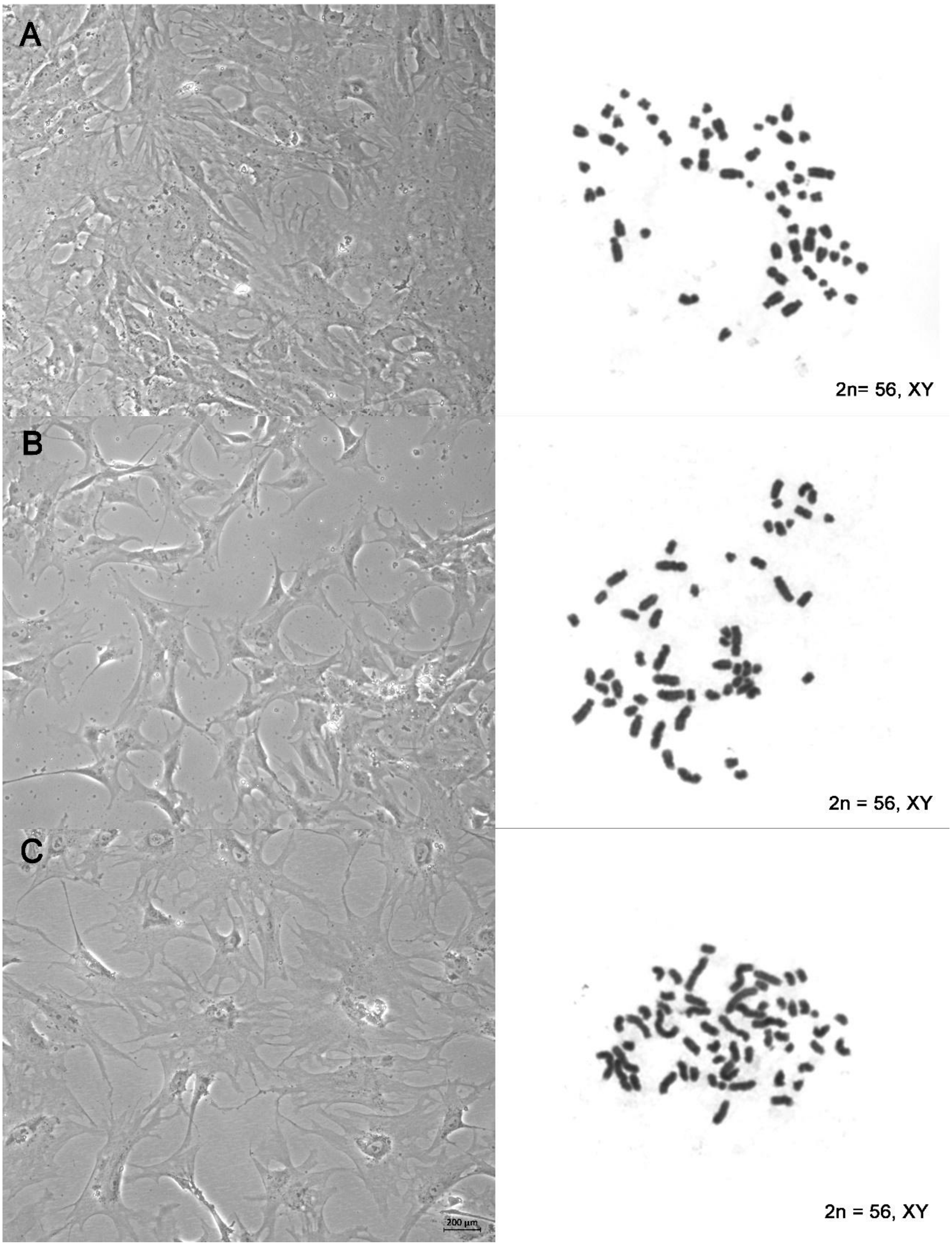

Figure S2: Images of Amazonian manatee (TINsf) cultures on the left and their respective karyotypes (2n=56, XY; FN=92) on the right: A) 3rd passage, B) 7th passage, and C) 12th passage. Scale bar: 200 µm.

Supplementary material S3:

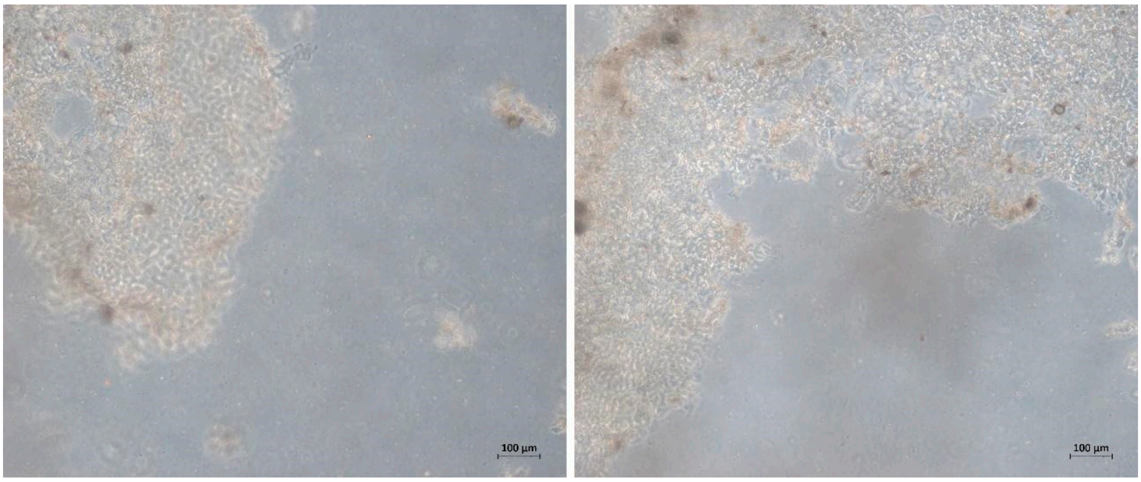

Figure S3: Images of TINsf cells exposed to a concentration of 5000 µg/mL of andiroba seed oil, which led to cell death within a few hours. Scale bar: 10 µm.
